# Case Report: Identification of a novel *NTRK3-AJUBA* fusion co-existing with *ETV6-NTRK3* fusion in papillary thyroid carcinoma

**DOI:** 10.3389/fonc.2023.1123812

**Published:** 2023-04-28

**Authors:** Qing-Xiang Yu, Wen-Jun Zhao, He-Yue Wang, Lei Zhang, Lan Qin, Lei Zhang, Jian-li Han

**Affiliations:** ^1^ Key Laboratory of Digital Technology in Medical Diagnostics of Zhejiang Province, Dian Diagnostics Group Co., Ltd., Hangzhou, Zhejiang, China; ^2^ Department of Thyroid & Bariatric Metabolic Surgery, Shanxi Bethune Hospital, Shanxi Academy of Medical Sciences, Third Hospital of Shanxi Medical University, Taiyuan, Shanxi, China; ^3^ Clinical Genome Center, Dian Diagnostics Group Co., Ltd., Hangzhou, Zhejiang, China

**Keywords:** papillary thyroid carcinoma, *NTRK3-AJUBA*, *ETV6-NTRK3*, NGS, fusion

## Abstract

*NTRK* fusions are validated oncogenic drivers of various adult and pediatric tumor types, including thyroid cancer, and serve as a therapeutic target. Recently, tropomyosin receptor kinase (TRK) inhibitors, such as entrectinib and larotrectinib, display promising therapeutic efficacy in *NTRK*-positive solid tumors. Although some *NTRK* fusion partners have been identified in thyroid cancer, the spectrum of *NTRK* fusion is not fully characterized. In this study, a dual *NTRK3* fusion was identified by targeted RNA-Seq in a 47-year-old female patient with papillary thyroid carcinoma. The patient harbors a novel in-frame fusion between *NTRK3* exon 13 and *AJUBA* exon 2, co-existing with a known in-frame fusion between *ETV6* exon 4 and *NTRK3* exon 14. The dual *NTRK3* fusion was validated by Sanger sequencing and fluorescence *in situ* hybridization (FISH) but lack TRK protein expression as defined by pan-TRK immunohistochemistry (IHC). We supposed the pan-TRK IHC result to be falsely negative. In conclusion, we present the first case of a novel *NTRK3-AJUBA* fusion co-existing with a known *ETV6-NTRK3* fusion in thyroid cancer. These findings extend the spectrum of translocation partners in *NTRK3* fusion, and the effect of dual *NTRK3* fusion on TRK inhibitor therapy and prognosis needs long-term follow-up.

## Introduction

Thyroid cancer is one of the most common malignant tumors, with papillary thyroid carcinoma (PTC) as the predominant subtype. The worldwide incidence of thyroid cancer in adults has been increasing dramatically in the past three decades, especially for PTC (1). According to the latest epidemiological research, PTC was the main contributor to the rapid increase in thyroid cancer incidence, and was the only histological subtype that increased systematically in 25 studied countries (2). Neurotrophic tyrosine receptor kinase (*NTRK*) fusions are validated oncogenic drivers of various adult and pediatric tumor types, including *NTRK1*, *NTRK2*, and *NTRK3*, which encode the TRK proteins TRKA, TRKB, and TRKC, respectively (3). Since the initial discovery in colorectal carcinoma (4), *NTRK* fusions have been identified in 17 unique cancer types, including thyroid cancer (5). *NTRK* fusions are found at high frequencies(>90%) in rare cancer types (secretory carcinoma, secretory breast carcinoma, infantile fibrosarcoma, and cellular or mixed congenital mesoblastic nephroma), moderate frequencies (5%-25%) in some cancers (papillary thyroid cancer, spitzoid neoplasm, and gastrointestinal stromal tumor) and lower frequencies(<5%) in other common tumors (lung cancer, breast cancer, colorectal cancer, pancreatic cancers, melanoma, and other solid or hematologic cancers) (5).

For structurally persistent/recurrent locoregional or distant metastatic disease not amenable to radioactive iodine (RAI) therapy, the National Comprehensive Cancer Network (NCCN) Guidelines recommend genomic testing to identify actionable mutations (including *NTRK* fusions). In several small basket trials, entrectinib and larotrectinib display promising therapeutic efficacy with high and durable responses in *NTRK* fusion-positive pediatric and adult solid tumors (6, 7). Although several *NTRK* fusion partners have been identified in thyroid cancer (8), the spectrum of *NTRK* fusion is not fully characterized. In this case, we first report a novel *NTRK3-AJUBA* fusion co-existing with *ETV6-NTRK3* fusion in PTC. We discuss the implications of this finding for targeted therapies and clinical outcomes.

## Results

### Clinical and pathological features

A 47-year-old female was initially present with a thyroid nodule during a routine physical examination in 2021. Additional complaints include anemia, leukocytopenia, and complete right bundle branch block (CRBBB). The patient reported that she had three siblings and had no family history of cancer. Ultrasonography revealed diffuse echogenic changes in the parenchyma of the right lobe with multiple punctate calcifications (Thyroid Imaging Reporting and Data System (TIRADS) 5), multiple nodules with punctate calcification in the right lobe (TIRADS 4a), and multiple enlarged lymph nodes in the right II-IV levels and VI level. She was then diagnosed with malignancy and underwent extended radical thyroidectomy on 6 September 2021. Postoperative pathological examination identified papillary thyroid microcarcinoma (3-mm maximum diameter) in the left lobe, multifocal PTC (range 0.6-1.2cm in diameter) in the right lobe, lymph node metastasis (2/7) in left central, lymph node metastasis (13/17) in right central, lymph node metastases in right level II (3/5), and lymph node metastases in right level III-V (5/8). She was treated with iodine-131 once after surgery. This patient had been followed up for 19 months and remained free of recurrence to date, she was satisfied with the treatment.

### Fusion description

**Figure 1:**
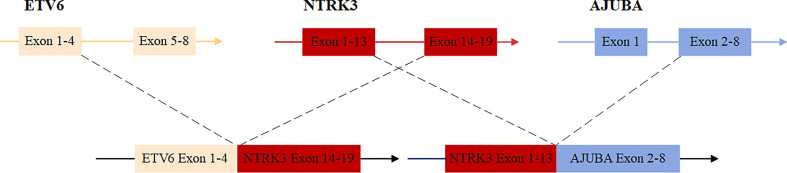
Schematic diagram of the novel nonreciprocal/reciprocal *NTRK3* translocation.

**Figure 2:**
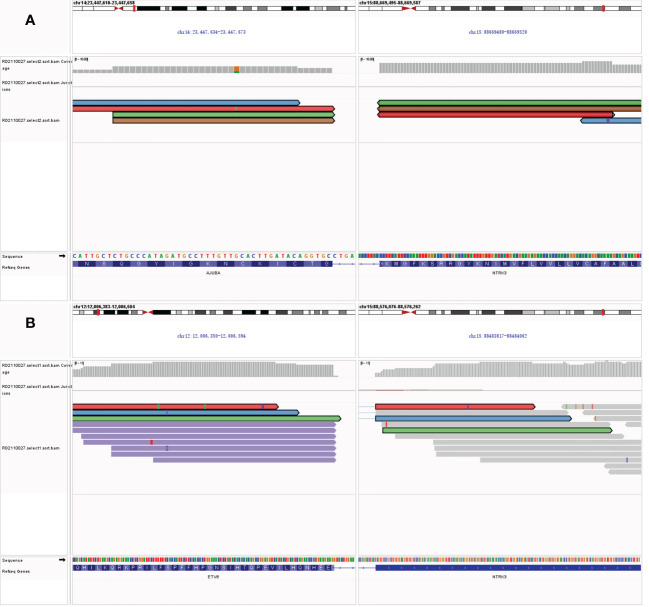
**(A)** Integrative Genome Viewer (IGV) snapshot of *NTRK3–AJUBA* fusion; **(B)** Integrative Genome Viewer (IGV) snapshot of *ETV6-NTRK3* fusion.

Freshly resected tumor tissue was used for gene fusion detection by targeted RNA sequencing, which was designed to target 22 genes frequently rearranged in thyroid carcinoma. Molecular testing revealed a novel fusion transcript between exon 13 of *NTRK3* and exon 2 of *AJUBA*, co-existing with a known fusion transcript between exon 4 of *ETV6* and exon 14 of *NTRK3* (**Figure 1**). The bioinformatics analysis detected 5 unique reads spanning the *NTRK3–AJUBA* fusion breakpoint and 95 unique reads spanning the *ETV6-NTRK3* fusion breakpoint. Meanwhile, we used Integrative Genome Viewer (IGV) to check and visualize the supporting reads that demonstrate the novel *NTRK3–AJUBA* fusion (**Figure 2A**) and *ETV6-NTRK3* fusion (**Figure 2B**), respectively.

**Figure 3:**
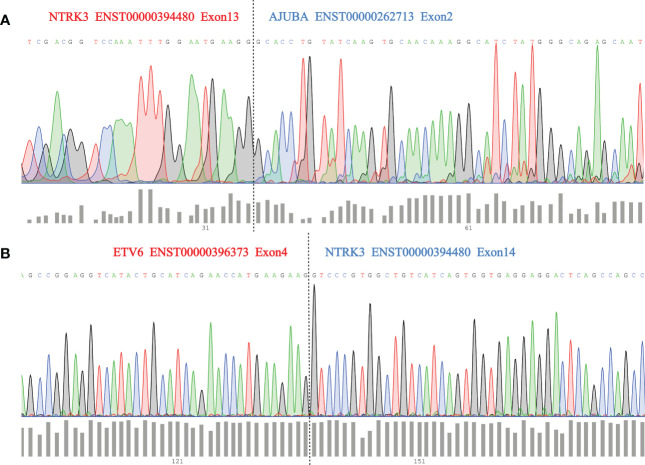
**(A)** Sanger sequencing validation of *NTRK3–AJUBA* fusion and dashed lines (black) indicate breakpoint position; **(B)** Sanger sequencing validation of *ETV6-NTRK3* fusion and dashed lines (black) indicate breakpoint position.

**Figure 4:**
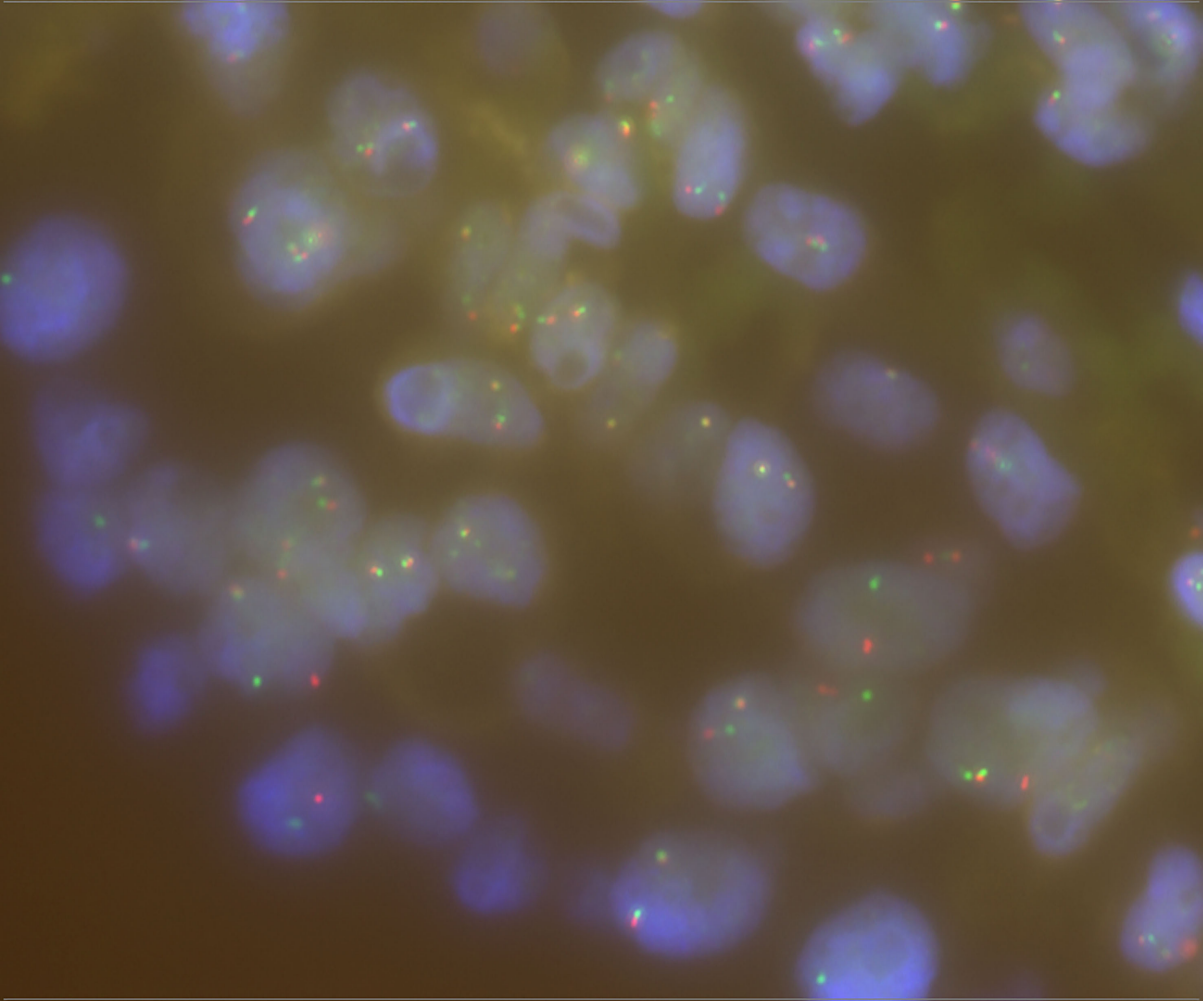
FISH break-apart assay for *NTRK3* gene and separate red and green signals indicating rearrangement.

We then used Sanger sequencing to validate the dual *NTRK3* fusion. The results revealed that exon 13 of the *NTRK3* gene on chromosome 15 is fused to exon 2 of the *AJUBA* gene on chromosome 14 (**Figure 3A**) and exon 4 of the *ETV6* gene on chromosome 12 is fused to exon 14 of the *NTRK3* gene on chromosome 15 (**Figure 3B**). Subsequently, fluorescence *in situ* hybridization (FISH) analysis, using a break-apart assay, confirmed *NTRK3* fusion at the genomic level (**Figure 4**). In addition, Pan-TRK IHC was performed to validate the presence of *NTRK* fusion protein. However, this case showed negative cytoplasmic and nuclear staining for pan-TRK (**Supplementary Figure 1**), which was discordant with NGS and FISH results.

The *NTRK3–AJUBA* fusion protein is predicted to include LRRNT, LRR_8, LRRCT_2, Ig, and I-set domains encoded by *NTRK3* and LIM domains encoded by *AJUBA*. The *ETV6-NTRK3* fusion protein is predicted to include the PNT domain encoded by *ETV6* and the whole protein tyrosine kinase domain encoded by *NTRK3* (**Supplementary Figure 2**).

## Discussion

In this report, we identified a dual *NTRK3* fusion in a 47-year-old female patient with PTC by targeted RNA sequencing. To the best of our knowledge, this is the first report of a novel *NTRK3-AJUBA* fusion co-existing with *ETV6-NTRK3* fusion in thyroid cancer or any other cancers. These findings extend the spectrum of translocation partners in *NTRK3* fusions. Although this case showed negative cytoplasmic and nuclear staining for pan-TRK IHC, the dual *NTRK3* fusion was detected by RNA-based NGS assay, and then validated by Sanger sequencing and FISH break-apart assay. Currently, there are no commercially available TRKC monoclonal antibodies (specific to *NTRK3* fusions), and some cases with *NTRK3* fusions showed negative results as defined by pan-TRK IHC (9, 10). Therefore, the Pan-TRK IHC result of this case may be falsely negative, and the RNA-based NGS assay was strongly recommended for *NTRK3* fusion detection. These findings provide evidence for the selection of *NTRK3* fusion detection methods and may contribute to precision diagnosis and treatment.

*ETV6* encodes an ETS family transcription factor and is located on chromosome 12. *NTRK3* encodes tropomyosin receptor kinase C (*TRKC*) and is located on chromosome 15. *ETV6-NTRK3* chimeric was a common oncogene fusion in a variety of cancers, including infantile fibrosarcoma (11), acute myeloid leukemia (12), mammary analogue secretory carcinoma (13), congenital mesoblastic nephroma (14), secretory breast carcinoma (15) and radiation-related PTC (16). Here, we describe a patient with papillary thyroid carcinoma harboring an *ETV6-NTRK3* fusion without radiation exposure and may benefit from TRK inhibitor therapy after possible tumor recurrence. *AJUBA* (Ajuba LIM Protein) gene functions as a scaffold participating in a variety of cellular processes, such as mitosis, motility, cell adhesion, gene transcription, cell differentiation, proliferation, and migration (17–20). Although numerous studies have demonstrated that *AJUBA* acts as an oncogene or tumor suppressor in different cancer types (19), the function of *AJUBA* in thyroid cancer remains unclear. Dysregulation of gene expression and gene mutations were the predominant genetic alteration in *AJUBA*, here we first reveal that *AJUBA* has undergone a gene fusion event, which is a new genomic alteration type in *AJUBA*.

This patient had been followed up for 19 months and remained free of recurrence or metastasis to date. The effect of nonreciprocal/reciprocal *NTRK3* fusion on TRK inhibitor therapy and prognosis needs long-term follow-up. To the best of our knowledge, nonreciprocal/reciprocal *NTRK3* fusion had not been reported before in thyroid cancer or any other cancers, possibly owing to the relatively low incidence of *NTRK* fusions in common tumors and the limitations of detection methods. Although the clinical significance of these nonreciprocal/reciprocal *NTRK3* fusions is still unknown, nonreciprocal/reciprocal *ALK* fusions had previously been reported to be associated with brain metastases and worse progression-free survival (PFS) in patients with *ALK*-rearranged NSCLC who received first-line crizotinib (21), moreover, nonreciprocal/reciprocal *ROS1* fusions may improve sensitivity to crizotinib and prolong PFS of patients with lung adenocarcinoma (22), indicating a potential role of the nonreciprocal/reciprocal *NTRK3* fusion in targeted therapy and prognosis of thyroid carcinoma.

## Materials and methods

### Targeted RNA sequencing and identification of gene fusions

The fusion gene panel was designed to target 22 genes frequently rearranged in thyroid carcinoma, including *ALK, BRAF, BRD4, DERL1, ERBB4, FGFR1, FGFR2, LTK, MAML2, MET, NTRK1, NTRK2, NTRK3, PAX8, PPARG, RAF1, RET, ROS1, SLC26A11, SLC5A11, THADA*, and *WNK1*. Nucleic acid extraction, library preparation, hybrid Capture, and NGS (Illumina NextSeq 500, San Diego, CA) were carried out by the Key Laboratory of Digital Technology in Medical Diagnostics of Zhejiang Province. FASTQ sequencing files were aligned to the human reference genome (UCSC hg19; Feb 2009 release) with BWA software. STAR-Fusion and Arriba software was used to detect gene fusions. The candidate gene fusion transcripts were reviewed and visualized in Integrative Genome Viewer (IGV, Broad Institute, version 2.1.2).

### Sanger sequencing

Direct Sanger sequencing of PCR product was performed to validate the dual *NTRK3* fusion. The *NTRK3-AJUBA* fusion transcripts were amplified using primer pairs as follows: primer forward: GTGTCCTGTTGGTGGTTCTCT and primer reverse: CAAAGCACTGGGTGTGGTAGA (product length 156bp). The *ETV6-NTRK3* fusion transcripts were amplified using primer pairs as follows: primer forward: TGTAAAACGACGGCCAGTTCTTTCCAGGTGATGTGCTCT and primer reverse: CAGGAAACAGCTATGACCAAGCAGATTCAGACCCACAG (product length 559bp).

### Fluorescence *in situ* hybridization

*NTRK3* break-apart FISH was performed on FFPE samples. The FISH signals were scored by evaluating 100 tumor cell nuclei per case. Tumor cells showing split signals were concluded to have *NTRK3* fusion. A threshold of 15% nuclei positive for a break-apart signal was considered positive for gene fusion.

### Immunohistochemistry

IHC staining for pan-TRK expression was performed on the Benchmark Ultra platform (Ventana Medical Systems, Tucson, AZ) with Optiview DAB IHC Detection Kit, using a commercially available pan-TRK assay (rabbit monoclonal antibody, clone EPR17341, Assay, RTU, Roche, Ventana).

## Data availability statement

The datasets presented in this article are not readily available because of ethical/privacy restrictions. Requests to access the datasets should be directed to the corresponding author.

## Ethics statement

The studies involving human participants were reviewed and approved by the ethics committee of Shanxi Bethune Hospital. The patients/participants provided their written informed consent to participate in this study. Written informed consent was obtained from the individual(s) for the publication of any potentially identifiable images or data included in this article.

## Author contributions

Conceptualization: Q-xY; validation: LQ; data curation: H-yW and LZ (4th author); writing—original draft preparation: Q-xY and W-jZ; writing—review and editing: LZ (6th author) and J-lH. All authors contributed to the article and approved the submitted version.
