# Supplementary material for: Case Report: Identification of a novel NTRK3-AJUBA fusion co-existing with ETV6-NTRK3 fusion in papillary thyroid carcinoma
Source: Front Oncol. 2023 Apr 28;13:1123812. doi: 10.3389/fonc.2023.1123812 (PMC10176450; doi:10.3389/fonc.2023.1123812)
Supplement: Supplementary file 1 [file Image_1.pdf]

Supplementary Material

Identification of a Novel *NTRK3-AJUBA* Fusion Co-existing with *ETV6-NTRK3* Fusion in Papillary Thyroid Carcinoma

Qing-xiang Yu[1†], Wen-jun Zhao[2†], He-yue Wang[2], Lei Zhang[2], Lan Qin[3], Lei Zhang[1*] and Jian-li Han[2*]
[1] Key Laboratory of Digital Technology in Medical Diagnostics of Zhejiang Province, No.329 Jin Peng Street，Xihu District，Hangzhou，Zhejiang Province，China
[2] Department of Thyroid & Bariatric Metabolic Surgery, Shanxi Bethune Hospital, Shanxi Academy of Medical Sciences, Third Hospital of Shanxi Medical University
[3] Dian Diagnostics Group Co., Ltd., No.329 Jin Peng Street, Xihu District，Hangzhou，Zhejiang Province, China

[*] **Correspondence:**
Lei Zhang: zhanglei3@dazd.cn; +86 18612084486;
Jian-li Han: 1292225922@qq.com; +86 13803456545
[†] These authors contributed equally to this work.

Supplementary Figures

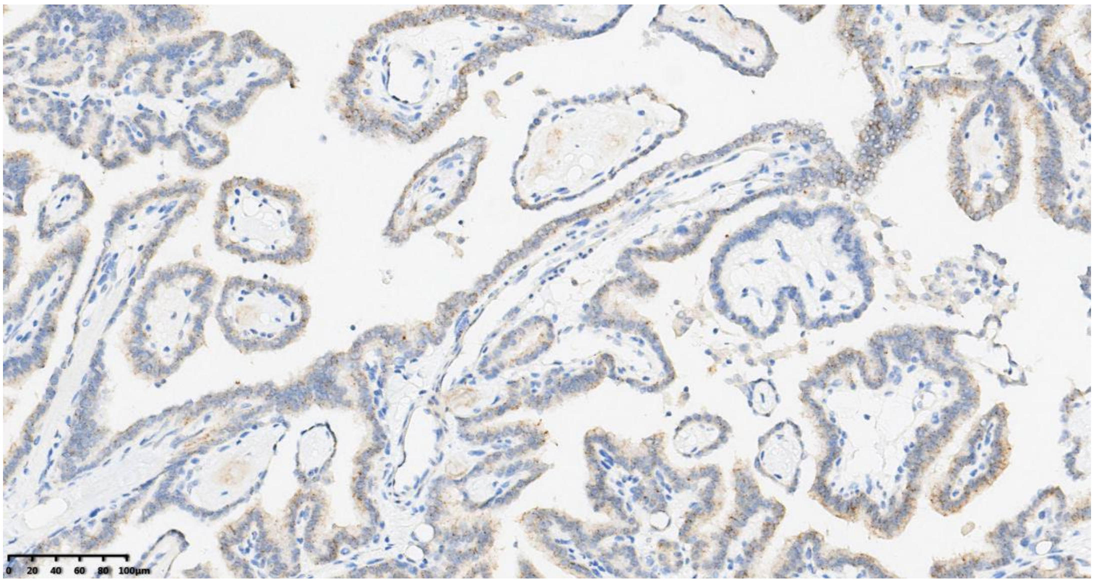

Supplementary Figure 1. Pan-TRK IHC with negative cytoplasmic and nuclear staining in PTC with a dual *NTRK3* fusion (20×).

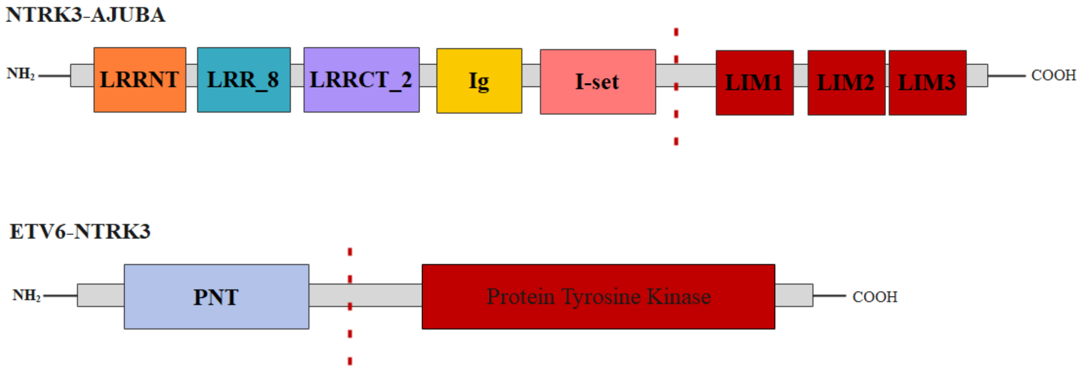

Supplementary Figure 2. Schematic diagram of the predicted fusion protein. The upper image shows the schematic diagram of the predicted *NTRK3–AJUBA* fusion protein, the lower image shows the schematic diagram of the predicted *ETV6-NTRK3* fusion protein. Abbreviations: LRRNT, Leucine-rich repeat N-terminal domain; LRR, Leucine-rich repeat domain; LRRCT, Leucine-rich repeat C-terminal domain; Ig, Immunoglobulin domain; I-set, Immunoglobulin I-set domain; LIM, LIM domain; PNT, Pointed domain.
